# Estimates of Future New Particle Formation under Different Emission Scenarios in Beijing

**DOI:** 10.1021/acs.est.2c08348

**Published:** 2023-03-17

**Authors:** James Brean, Alex Rowell, David C. S. Beddows, Zongbo Shi, Roy M. Harrison

**Affiliations:** † School of Geography, Earth & Environmental Sciences University of Birmingham, Birmingham B15 2TT, United Kingdom; ‡ Department of Environmental Sciences, Faculty of Meteorology, Environment and Arid Land Agriculture, King Abdulaziz University, Jeddah 21589, Saudi Arabia

**Keywords:** NPF, nucleation, growth, aerosols, net-zero, China

## Abstract

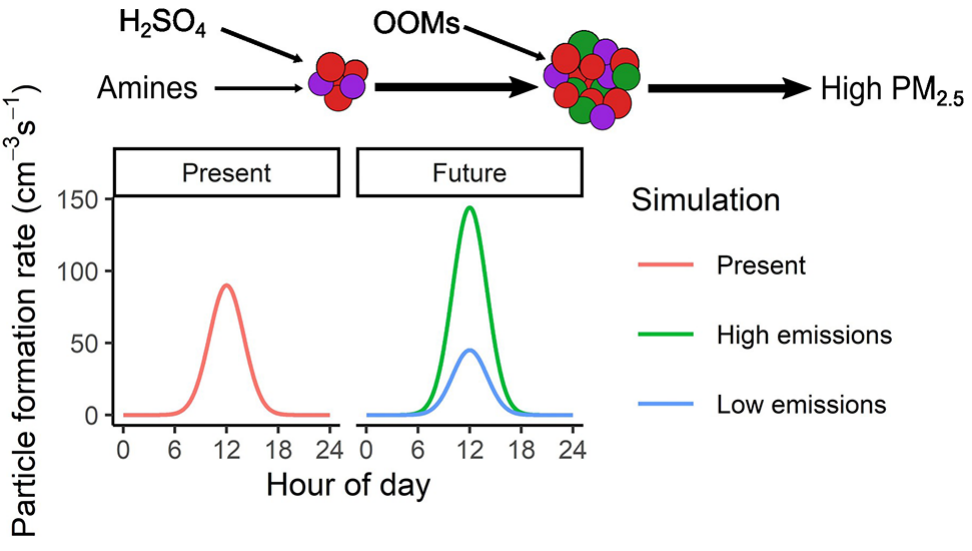

New particle formation (NPF) is a leading source of particulate matter by number and a contributor to particle mass during haze events. Reductions in emissions of air pollutants, many of which are NPF precursors, are expected in the move toward carbon neutrality or net-zero. Expected changes to pollutant emissions are used to investigate future changes to NPF processes, in comparison to a simulation of current conditions. The projected changes to SO_2_ emissions are key in changing future NPF number, with different scenarios producing either a doubling or near total reduction in sulfuric acid-amine particle formation rates. Particle growth rates are projected to change little in all but the strictest emission control scenarios. These changes will reduce the particle mass arising by NPF substantially, thus showing a further cobenefit of net-zero policies. Major uncertainties remain in future NPF including the volatility of oxygenated organic molecules resulting from changes to NO_*x*_ and amine emissions.

## Abstract 2

New particle formation (NPF) is a key source of atmospheric particles. Here, we estimate future changes to particles derived from NPF and show it to be sensitive to future pollutant emissions.

## Introduction

Air pollution and climate change influence many aspects of our societies. The former results in pollution-related mortality^1,2^ and economic impacts such as reduced agricultural yields,^3^ while the latter threatens many aspects of our earth systems.^4^ Efforts to combat emissions of climate forcing agents will come with air quality cobenefits,^5^ for example, black carbon is both one of the most important climate forcers and is a significant contributor to global PM_2.5_ mass and thus PM_2.5_ related morbidity and mortality. The mortality cobenefit related to greenhouse gas (GHG) emission reduction is estimated to be 1.3 ± 0.5 million premature deaths avoided by 2050,^6^ with efforts to reduce short-lived climate pollutants having the most immediate effect.^7^ These estimates are dependent on the emission reduction scenario and the consequent future impact of emission reduction policies on the composition of urban air and relevant atmospheric processes.

To limit global climate change, many countries have committed to a net-zero emissions target, albeit on different time horizons. Net-zero depends upon a balance between the amount of GHG emissions produced by anthropogenic activities and the amount removed from the atmosphere over a specified period. This is proposed to be achieved by a combination of emission reduction strategies and active removal processes. The consequent changes in atmospheric composition are expected to impact new particle formation (NPF).

NPF is an important atmospheric process wherein gas phase molecules cluster together and grow to form new aerosol particles. These aerosol particles have the potential to contribute greatly to PM_2.5_ mass loadings,^8−10^ thereby reducing regional air quality. Kulmala et al.^10^ show that, under conditions typical for Beijing, a new mode of freshly formed particles will have a mass of 10 μg m^–3^ after a little over 12 h and >100 μg m^–3^ after 48 h. NPF also contributes heavily to global cloud condensation nuclei budgets,^11^ providing great uncertainty in estimates of global radiative forcing. NPF can be usefully conceptualized as a two-step process where first gases cluster together (particle formation), and second, these clusters grow to larger sizes by condensation of vapors or coagulation with other particles (particle growth). The growth rate (GR) of new particles is a key parameter for particle survival in polluted environments as the loss rates due to coagulation of such particles are high;^12,13^ thus, future changes to particle GR will have a substantial effect on the future yields of particles from NPF. While present-day NPF is well-understood,^14^ modeling studies of preindustrial^11,15^ and future^15−17^ NPF are sparse. Future studies predict decreases in aerosol radiative forcing,^15,16^ total particle number,^17^ or sulfate aerosol^18,19^ due to reduced future anthropogenic SO_2_, although such studies use relatively simple NPF parametrizations and all neglect amines as stabilizing species.

In the urban environment, particle formation from sulfuric acid and amines has been shown to be the dominant mechanism,^20−23^ and thus, the new particle formation rate (J) in these environments is dependent on the abundance of sulfuric acid and base molecules and the loss rates by coagulation and evaporation. Particle growth is primarily driven by condensation of vapors, a process limited by the size of the particles and the volatility and abundance of the vapor molecules. Condensation of sulfuric acid and oxygenated organic molecules (OOMs) can largely explain particle growth observed in the urban environment.^20,21,24^ Particle loadings produced by NPF are therefore sensitive to gas-phase precursor concentrations, oxidant concentrations, pre-existing particle surface area loading, and ambient temperatures. In Beijing, the condensation of anthropogenic OOMs on new particles has been shown to drive particle growth^24^ and condensation onto particles of all sizes has been shown to be a major contributor to secondary organic aerosol loadings.^25^

In this study, we investigate the possible future changes to NPF. We use a zero-dimensional cluster and aerosol dynamic box model that simulates present-day formation and growth in Beijing (see the Materials and Methods section), where processes contributing to particle formation and growth are well-characterized.^8−10,20,23,24,26^ We use future predictions for the effect of the net-zero policy on the emissions of SO_2_, volatile organic compounds (VOCs), NH_3_, amines, NO_*x*_, and particulate matter (PM) to investigate the possible future changes to NPF in China (Beijing) under a range of different climate constraints, socioeconomic drivers, and air pollution control measures. We probe the changes to resultant particle mass from NPF under these scenarios and show that emission reductions will come with the cobenefit of reduction to secondary particle mass from NPF.

## Materials and Methods

### Estimating NPF

We constructed a zero-dimensional cluster and aerosol dynamic box model to simulate new particle formation and growth (see the Supporting Information for details of calculations). The box model simulates the formation of sulfuric acid and OOMs from oxidation of SO_2_ and a proxy VOC by OH and O_3_. The OOM yield is 2%, in line with yields from a range of VOCs.^27^ NPF occurs regionally across Beijing, and transport plays a relatively minor role in affecting the size distribution; thus, a zero-dimensional model is appropriate here.^28^ New particles with four acid and four base molecules are produced from sulfuric acid and a base molecule with the same properties as dimethylamine (DMA), accounting for collisional formation, and losses due to evaporation and coagulation into larger particles.^26^ The rate of formation of these particles is dubbed J_A4B4_. Formation rates are thus sensitive to temperature^29^ and condensation sink (CS). CS is the rate at which a molecule in the gas phase, here, presumed as sulfuric acid, will be lost to pre-existing particle surface due to condensation. In Beijing, CS is a major determinant of NPF occurrence.^30^ Growth of molecules is due to both condensation of acid and base clusters and OOMs and coagulations of new particles.^24^ Particles exist in 100 logarithmically spaced bins between 1.5 and 2500 nm. The concentrations and diurnal profiles of these species and the resultant formation and growth rates of new particles were tuned to be similar to those in Beijing. The volatility distribution of OOMs was taken to be the same as that observed in Beijing,^24^ and the mean concentration of OOMs is similar to that reported for Beijing (1.2 × 10^8^ cm^–3^), where Qiao et al.^24^ report summertime concentrations of 4.0 × 10^8^ cm^–3^. All simulations were performed at 293 K and 50% RH. The model runs through 1440 1 min timesteps for 24 h.

### Emission Scenarios

We combined our NPF-focused assessment model with a technology-based emission projection model (Dynamic Projection for Emission in China, DPECv1.1)^33,34^ to investigate the possible future changes to NPF in China (Beijing) under a range of different climate constraints, socioeconomic drivers, and air pollution control measures developed by Tsinghua University. The DPEC model was used to generate total emission estimates for NH_3_, SO_2_, VOCs, and NO_*x*_ in Beijing for the base year (2020) and future years (2040/2060). Total emissions were used instead of pollutant concentrations due to the availability of data. However, pollutants with relatively short atmospheric lifespans are tightly coupled with emission rates, and therefore, fluctuations are reflected in their concentrations.^35^ The scenarios used in this study are detailed below and summarized in Table 1, and changes to emissions relative to 2020 are in Table 2. Further documentation and access to the DPEC model is available at http://meicmodel.org.

1. Baseline provides a point of comparison for the other scenarios in this paper. It follows the SSP4 narrative, which envisions slower economic growth and a fast-increasing population. Climate constraints are minimal under RCP6.0 conditions and air pollution controls remain at 2015 levels.
2. Current Goals presumes China will achieve its National Determined Contribution (NDC) pledges by 2030. It follows the SSP2 narrative, which envisions slow economic progress. Climate constraints are moderate under RCP4.5 conditions and air pollution controls adopt current and upcoming policy to 2030.
3. Ambitious-pollution-Neutral Goals represents China’s commitment to achieve carbon neutrality by 2060. It follows the SSP1 scenario, which envisions a gradual shift toward a more sustainable society. Climate constraints are more ambitious under China’s carbon neutral goals and air pollution controls adopt the best available end-of-pipe technologies.

**Table 1:** Relative Changes in Emissions for Various Scenarios within Dynamic Projection of Anthropogenic Emissions in China Model for Beijing

year	scenario	NH_3_	SO_2_	VOCs	NO_X_	PM_2.5_
2020	Baseline	1	1	1	1	1
2040	Baseline	1.09	2.05	1.11	1.46	1.61
	Current Goals	0.90	0.64	0.67	0.55	0.90
	Ambitious-pollution-Neutral Goals	0.79	0.18	0.41	0.34	0.21
2060	Baseline	1.19	2.00	1.04	1.53	1.54
	Current Goals	0.92	0.55	0.62	0.58	0.81
	Ambitious-pollution-Neutral Goals	0.71	0.02	0.25	0.15	0.18

**Table 2:** Summary of Dynamic Projection of Anthropogenic Emissions in China Scenarios Used in This Study^a^

scenario	policy constraints	socio-economic drivers	air pollution control measures	years
Baseline	RCP6.0	SSP4	business-as-usual	2020, 2040, and 2060
Current Goals	RCP4.5	SSP2	enhanced-control-policy	2040 and 2060
Ambitious-pollution-Neutral Goals	neutrality	SSP1	best-health-effect	2040 and 2060

a Detailed scenario analysis is available from Tong et al.^31^ and Cheng et al.^32^

For brevity, these shall be referred to as the “baseline”, “current”, and “ambitious” scenarios, respectively.

## Results and Discussion

### Characteristics of Simulated Future and Present NPF

**Figure 1:**
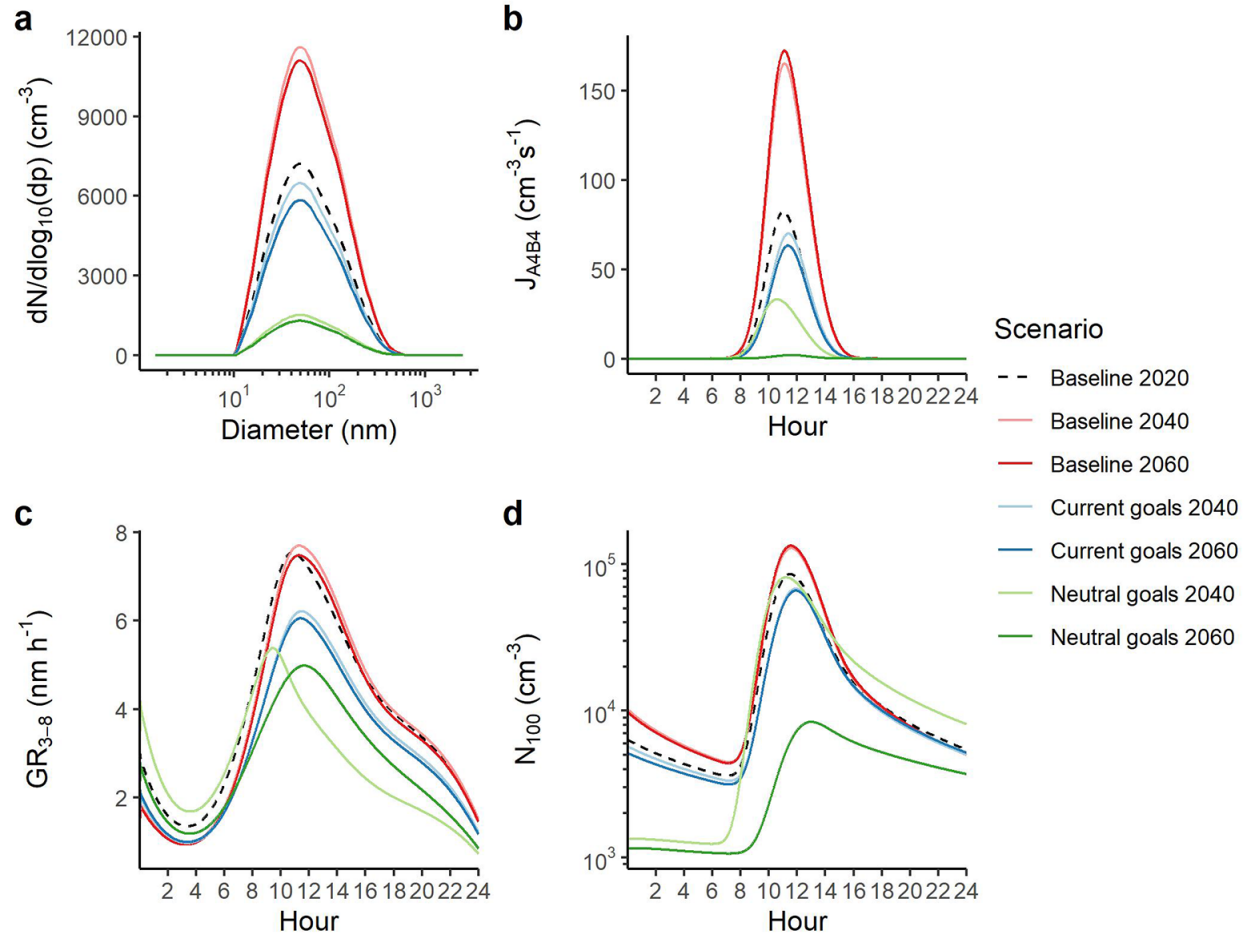
NPF estimates for the seven different scenarios, showing (a) the initial size distribution of particles at the start of each simulation run, (b) the diurnal cycle of J_A4B4_, (c) the diurnal cycle of GR_3–8_, and (d) the diurnal cycle of N_100_.

Our simulation results are plotted in Figure 1. Figure 1a shows the initial size distribution, the average of TSI LongSMPS data taken in Beijing.^36^ Condensation sink (CS) is the rate at which a molecule, here, sulfuric acid, in the gas phase will be lost to pre-existing particle surfaces due to condensation. In Beijing, CS is a major determinant of NPF occurrence.^30^ Our initial size distribution corresponds to an initial CS of 0.013 s^–1^. A recent analysis of new particle formation and growth in Beijing shows, while highly variable, the formation rate of particles at 1.5 nm (*J*_1.5_) has a median annual value of 79 cm^–3^ s^–1^ and a springtime median of ∼40 cm^–3^ s^–1^.^22^ Our present-day simulation has a peak in the formation rate of particles with four acid and four base molecules (J_A4B4_) of 82 cm^–3^ s^–1^, and a mean value across the NPF event of 35 cm^–3^ s^–1^, defining the NPF event as the period where JA4B4 is greater than 1 cm^–3^ s^–1^, accurately simulating real-world particle formation rates. Real-world measurements of GRs give annual medians of 0.9, 1.7, 2.8, and 2.9 nm h^–1^ in the size ranges 1–3, 3–7, 7–15, and 15–25 nm.^22^ Our simulation gives GRs of 3.1, 3.9, 4.3, and 4.3 nm h^–1^ in these size ranges, different from measurement data but similar to chamber results for these concentrations of OOMs.^37^ Our higher growth rates in smaller sizes are driven by condensation of organics.

**Figure 2:**
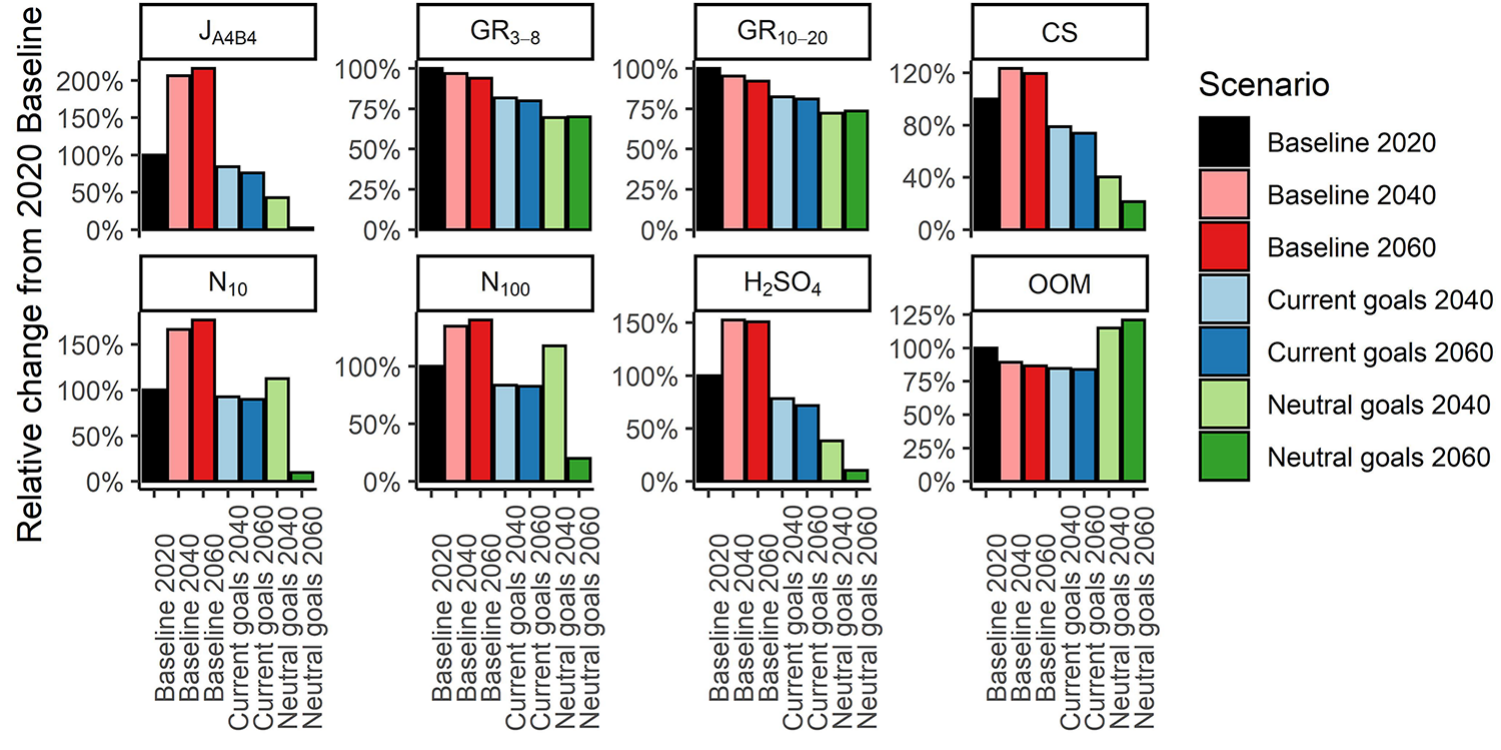
Changes in mean particle concentrations <10 nm and <100 nm: J_A4B4_, CS, GR_3–8_, GR_10–20_, [H_2_SO_4_], and [OOM] relative to the “Baseline 2020” scenario.

Future scenario results are also plotted in Figure 1, and changes to NPF relative to present day results are shown in Figure 2. Long-term analyses have shown the PM_2.5_ mass to correlate well with CS; therefore, we scale future CS with estimated PM_2.5_.^30^ These different initial size distributions correspond to CSs of 0.013, 0.021, and 0.020 s^–1^ in the Baseline 2020, 2040, and 2060 scenarios, 0.012 and 0.011 s^–1^ in the Current Goals 2040 and 2060 scenarios, and 0.003 and 0.002 s^–1^ in the Ambitious 2040 and 2060 scenarios. The Baseline 2040 and 2060 scenarios therefore begin with a higher CS than the Baseline 2020, and the Current Goals and Neutral Goals scenarios begin with a lower CS than the Baseline 2020 one.

In the box model, J_A4B4_ is dependent on H_2_SO_4_ and base concentrations. The box model reproduced chamber observations of nucleation rates (Figure S2). J_A4B4_ decreases with increasing temperature and CS (Figure S2); however, for these simulations, temperature is kept constant at 293 K. [H_2_SO_4_] is dependent on future changes to emissions of SO_2_ and changes to the condensation sink. As there are no predicted emissions in the present nor future for amine concentrations, amine emissions are presumed to scale with changes to NH_3_ emissions. The resultant J_A4B4_ values peak at midday, coincident with peak photochemistry (Figure 1b). For the Baseline cases, which represent a business-as-usual scenario, a 10–20% increase in amine concentrations and a doubling in SO_2_ result in an over 100% increase to J_A4B4_ across an NPF event, with J_A4B4_ peaking at 173 cm^–3^ s^–1^ for the Baseline 2060 scenario, outcompeting increases to CS. For the Current Goals, a 10% reduction in amine concentrations and a near halving in SO_2_ is expected by 2060. This results in a 16% and 23% decrease in peak J_A4B4_ by 2040 and 2060, respectively, despite lower CS values. Large reductions in SO_2_ are expected for the Neutral Goals scenario, with SO_2_ dropping by 82% and 98% by 2040 and 2060, respectively, with a 21% and 29% reduction to amines. This results in large decreases to J_A4B4_ values of 60% and 98%. With such a large reduction in J_A4B4_, new particles are rapidly lost to coagulation.

GRs are dependent on the OOM, sulfuric acid, and base concentrations in order of decreasing importance (Figure S3). Initial growth is driven by sulfuric acid and the least volatile OOMs. The more volatile OOMs with greater abundancy drive later stage growth as the Kelvin effect decreases at greater diameters. The concentrations of the OOMs are dependent on the VOC concentrations and the CS. The saturation mass concentration affects the efficiency with which OOM can condense onto particles, especially for those small sizes where the Kelvin effect is large (see eqs 10–14). GR_3–8_ has been calculated, as growth rates <10 nm are key in determining particle survival probability;^13^ however, measurements of growth rates <3 nm are highly uncertain. The diurnal variation of these GRs (Figure 1c) is driven by O_3_ and OH concentrations, peaking at midday before trailing off toward the afternoon. The Baseline 2040 and 2060 scenarios predict a small decrease in [OOM] of ∼10%, with an increase in [H_2_SO_4_]. This results in little change to GR_3–8_. Decreases in VOC emissions and CS in the Current Goals scenarios result in a ∼15% decrease in [OOM] and a ∼20% decrease to GR_3–8_ in 2040 and 2060. The Neutral Goals 2040 and 2060 scenarios predict an increase to [OOM] and a substantial decrease to [H_2_SO_4_], resulting in decreases to GR_3–8_ of ∼30%.

Both the pre-existing particle surface area and the J_A4B4_ and GR influence the resultant particle number concentrations (N_10_ and N_100_), which initially trail downward due to coagulation in the absence of particle formation (with a maintenance in particle volume) until midday, where particle formation causes a factor of 2–10 increase in particle number (scenario dependent, Figure 1d). Rapid losses of particles of diameters <10 nm due to coagulation and particle growth mean that average daily particle number concentrations with diameters <10 nm (N_10_) are similar to average daily J_A4B4_. Relative to the Baseline 2020 scenario, the peak in N_100_ increases by 65 and 75% in the Baseline 2040 and 2060 scenarios, respectively, reduces by 10% and 13% in the Current Goals 2040 and 2060 scenario respectively, shows no change in the Neutral Goals 2040 scenario, and decreases to near-zero in the 2060 scenario.

A sensitivity test to the particle counts in the initial particle number size distribution, and thus initial CS, is shown in Figure S4 showing a moderate effect on J_A4B4_ and GR with a ±50% multiplication to initial particle count. However, when the initial CS is increased by an order of magnitude (0.13 s^–1^), NPF is nearly wholly suppressed, and GRs are substantially reduced. Analysis of 1 year’s field data from Beijing shows that NPF mostly occurs when CS is <0.03 s^–1^, and no NPF occurred when CS was >0.1 s^–1^.^30^ A single order of magnitude reduction in CS (0.0013 s^–1^) results in little elevation to J_A4B4_ but a substantial increase to the NPF derived particle count relative to the background due to inefficient coagulational scavenging of new particles.

Figure S5 shows that shifting temperature has little effect on GRs as in chamber observations^38^ but drastically affects J_A4B4_ and therefore N_100_ due to the change in cluster evaporation rates. Temperature is maintained at 293 K throughout all simulations, which is in-line with the mean Beijing summertime temperature. Figure S6 shows the sensitivity of the box model to changing [OOM] concentrations. GR_3–8_ increases with increasing [OOM]. Increases in [OOM] and therefore GR cause an elevation to CS, reducing J_A4B4_ (mean J_A4B4_ is reduced by >50% due to a doubling in [OOM]). Decreases to [OOM] and therefore GR cause a decrease to CS, increasing J_A4B4_ (mean J_A4B4_ is increased >50% due to a halving in [OOM]). In the case of zero [OOM], GR_3–8_ is <1 nm h^–1^, and particles are rapidly lost to background aerosol; thus, N_100_ rapidly decreases after midday in this scenario. Figure S7 shows the effect of changing [SO_2_] and therefore [H_2_SO_4_]. Changes in [SO_2_] have small effects on GR and large effects on J_A4B4_. This translates to a drastic change to N_100_. In the case of a complete reduction in [SO_2_], no particle formation takes place. We presume that a sufficiently large (>50%) reduction to NO_*x*_ emissions will change the volatility distribution of OOMs by shifting them down by one decade in C*. Although NO*_x_* will ultimately impact yields of OOMs,^27^ the exact change in yield is uncertain. Here, the main determinants on OOM concentrations are VOC concentration and CS, with no effect from changing NO*_x_* on yield.

Our Baseline 2020 scenario therefore simulates present-day particle formation rates in Beijing accurately^22^ through a method consistent with modeling of chamber studies (Figure S2). The mean concentration of H_2_SO_4_ between 9:00–17:00 (9 × 10^6^ cm^–3^) is the same as that reported by Deng et al.^22^ during NPF events in the summertime. Growth rates due to H_2_SO_4_ and OOMs are similar to those seen in summertime Beijing by previous studies in all but the smallest size ranges, where we overestimate growth rates.^24^ This will serve to counterbalance the unrealistically low survival probabilities of <5 nm clusters found from ambient measurements.^10^ The mean concentration of OOMs is similar to that reported for Beijing (1.2 × 10^8^ cm^–3^), where Qiao et al.^24^ report summertime concentrations of 4.0 × 10^8^ cm^–3^. Growth of particles due to condensation of other inorganic species (i.e., HNO_3_)^13^ is neglected here. Condensation of sulfuric acid, its clusters, and OOMs is generally sufficient to explain particle growth in Beijing,^24^ although GRs do not necessarily correlate with [OOM] across a whole year.^10^

In our Baseline 2040 and 2060 scenarios, representing a “business as usual” future, increases to SO_2_ emissions have a greater effect than our predicted increases to CS, resulting in an elevation to particle number counts caused by NPF due to elevated J_A4B4_. This CS will result in little change in [OOM] and therefore GRs. In our Current Goals 2040 and 2060 scenarios, reductions in both CS and SO_2_ simultaneously lead to a small decrease in [H_2_SO_4_] and [OOM] and therefore small decreases in J_A4B4_ and GRs. Increased survival probabilities of small particles due to reduced coagulation sinks, however, result in a small increase to particle counts during NPF. Substantial decreases to SO_2_ and VOCs are predicted in the Neutral Goals 2040 and 2060 scenarios. This results in drastic decreases to [H_2_SO_4_] and [OOM], again outcompeting the reductions in CS. J_A4B4_ and GRs decrease, and thus, particle counts during NPF are reduced substantially in these scenarios (Figures 1 and 2).

Kulmala et al.^10^ show from long-term observations that the growth rate of particles in Beijing is not correlated with the number concentration of condensable material, defined as the highly oxygenated organic molecule (HOM) and sulfuric acid concentrations summed. They propose that either multiphase chemical reactions assist the growth rate or higher volatility compounds condense onto these particles. As the exact mechanism is unknown, we simply observe the condensation of OOMs as defined in Qiao et al.,^24^ which includes all organic molecules measured using nitrate chemical ionization mass spectrometry. This will include molecules more volatile than those, which fall into the category of HOM^27^ and produce growth rates consistent with those seen in Beijing. We also neglect hypothesized mechanisms of growth from ammonium nitrate,^13^ for example. Thus, while our simulations reproduce present-day particle growth rates, future dependence of particle growth on changes in VOC emissions may be overestimated.

### Mass of NPF-Derived Particles

**Figure 3:**
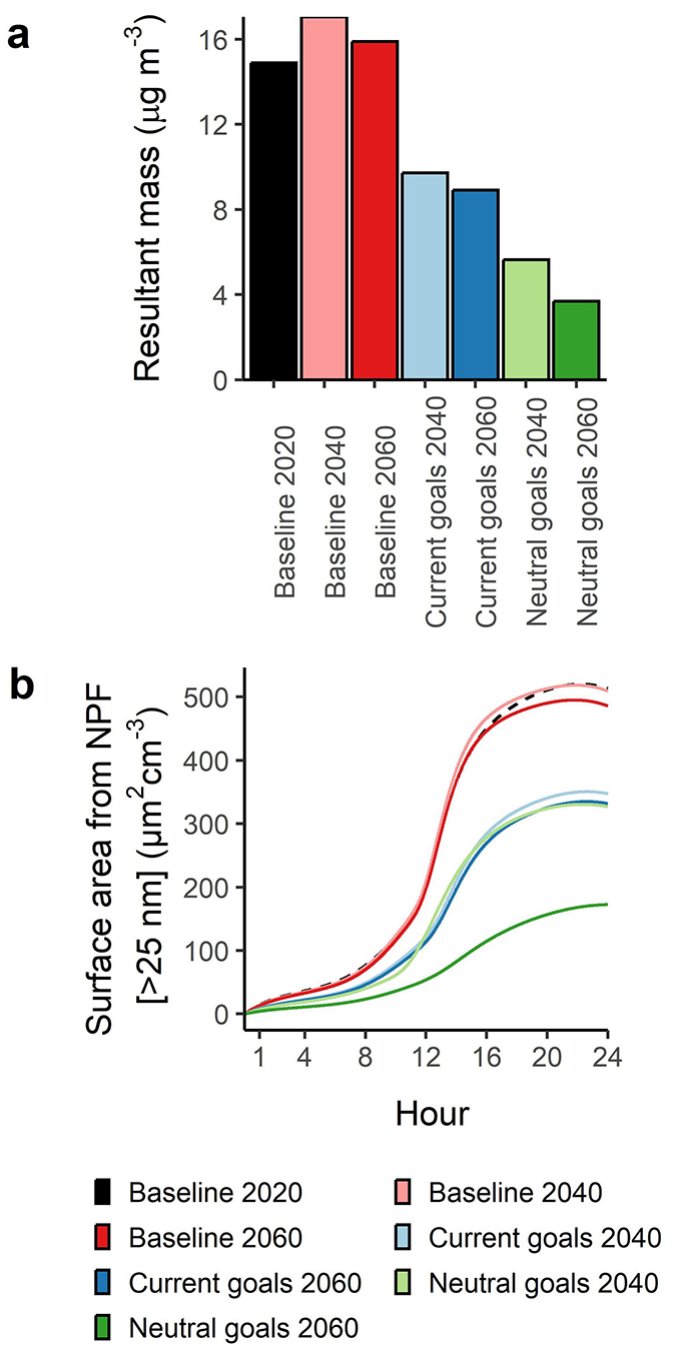
(a) Resultant particle masses in the box model after a 24 h NPF run minus the data from a run with particle formation and vapor condensation turned off, showing for each scenario total mass of particles, presuming a density of 1500 kg m^–3^. (b) Surface area of particles >25 nm arising from particle formation and vapor condensation.

We provide an estimate for the total particle mass arising from the formation of new particles and the condensation of low volatility vapors on both new and pre-existing particles (at the 1440th minute in the simulation) under different future emission scenarios by taking the difference in particle mass from simulations both with, and without particle formation and growth taking place (Figure 3a). The mass is measured approximately 12 h after the peak in J_A4B4_. Coagulation still takes place in both instances. Presuming an ideal NPF event with no cessation due to changing air masses, rain, dilution, or increasing CS due to emissions, we present the enhancement in particle mass due to particle formation and consequent condensation in Figure 3a. Particle formation enhances the condensation of OOMs greatly, as alongside subsequent growth in the Baseline 2020 case, it enhances total particle surface area by up to 35% relative to a simulation with no particle formation. In all scenarios, the contribution to total particle mass ranges from 4 to 17 μg m^–3^. These particle mass concentrations 12 h after the peak in NPF intensity (midday) are consistent with recent work by Kulmala et al.^10^ This contribution decreases in these scenarios with decreasing J_A4B4_ and GR, with the former showing greater variability between scenarios as seen in Figure 3. In the Baseline 2040 and 2060 cases, there is an increase to derived particle mass to 17 and 16 μg m^–3^, respectively. Under the Current Goals scenarios, secondary particle mass enhancement decreases by 40% to ∼9 μg m^–3^ for both 2040 and 2060, while under the stricter Neutral Goals 2040 and 2060 scenarios, this secondary particle mass reduces by 42% and 75% for the 2040 and 2060 scenarios, respectively, to 6 and 4 μg m^–3^, respectively. The structure, mass transport kinetics, and heterogeneous chemical reactions at the surface of particles will be distinct from those in the bulk.^39,40^ The highly oxygenated organic content of new particles in Beijing results in a high hygroscopicity,^41^ and thus, they will have a liquid water interface at the surface. Soluble organic material will dissolve in this surface more readily than in other particles,^42,43^ with subsequent reactions producing highly oxygenated material in the particle phase,^43^ which will itself be hygroscopic.^44^ The growth of particles greater than 25 nm in Beijing does not have a size dependence,^9^ indicating that, at these diameters and above, such surface uptake may dominate the growth process.^45^ The exact chemical mechanism is unknown and not modeled in this study, focusing only on condensation of OOMs; however, we show that net-zero policy will come with substantial decreases to particle surface area of particles >25 nm arising from this condensation in Figure 3a and, thus, a decrease in the hygroscopic and chemically active surface of new particles. The hygroscopic particles provide an enhanced surface area for uptake of gases and a medium for their oxidation, thus enhancing the mass of secondary aerosol beyond that resulting from gas phase oxidation processes alone.

NPF in Beijing has been shown to precede haze events.^8^ Recent calculations by Kulmala et al.^9^ and Kulmala et al.^10^ show a potential contribution from new particles to the mass of particles in the atmosphere of Beijing, while field observations across China show that anthropogenically derived OOMs are key to secondary aerosol formation.^25^ We show that future emission reductions will likely come with the cobenefit of reductions to secondary particle mass, while further increases in a “business as usual” case will lead to a small increase in the secondary particle mass. This secondary mass is seen to be substantial (Figure 3), increasing with greater J_A4B4_ and GR and is in-line with predictions from Kulmala et al.^10^ We therefore propose that the currently anticipated reductions in SO_2_ will play a key role in reducing NPF intensity and therefore potentially secondary particle mass in Beijing. More ambitious goals in the move toward net-zero will only hasten this cobenefit, reducing secondary PM_2.5_ mass concentrations. Further, as J_A4B4_ is highly dependent on the base concentrations, reduction of amine concentrations would also result in a reduction to J_A4B4_; although, as current amine sources and concentrations are poorly understood, this is more difficult to simulate. Our scenarios account for at most a 30% decrease to amine concentrations, but substantial decreases may result in different, less efficient bases acting as the primary base with which H_2_SO_4_ clusters during NPF. Due to the short lifetime of the NPF precursor gases and NPF-derived particles relative to GHGs, these air-quality cobenefits would be near immediate.

### Drivers of Change

The DPEC model contains emission contributions from the power, industrial, residential, transportation, and agricultural sectors for pollutant species. These include fossil fuel and biomass combustion, coke, steel and iron production, cement plants, petrochemical production emissions, solvent use, and agriculture. The relative importance of these sectors varies over time as emissions are curtailed and abated with the latest technology.

Our NPF simulations are highly dependent on SO_2_ emissions. In DPEC, the residential sector is responsible for the largest share of SO_2_ emissions in the Baseline and Current Goals scenarios in 2020, 2040, and 2060, increasing in the Baseline and decreasing in the Current Goals scenarios. In the Neutral Goals scenario, the power and industrial sectors dominate SO_2_ emissions in 2040 and 2060. By 2060, all trail down to near-zero. The industrial sector is accountable for the largest share of VOC emissions in all scenarios, with substantial emissions from residential sectors also, and changes to emissions from these sectors are the main drivers behind the projected changes in VOC emissions. The agricultural sector is answerable for the largest share of NH_3_ emissions in the Baseline, Current Goals, and Neutral Goals scenarios from 2020 through 2060, and changes to agricultural emissions are the main driving force behind the projected changes in NH_3_ emissions, with changes in contributions from the residential sector also playing a key role. The transportation sector produces the largest share of NO_*x*_ emissions in the Baseline scenario in 2020, 2040, and 2060. Reductions to transport emissions are the main driving force behind the projected fall in NO_*x*_ emissions in the Current Goals and Neutral Goals scenarios, and by 2040, industry takes over as the most significant sectoral source of NO_*x*_.

The main driver in change to NPF derived mass in our simulations is the change to SO_2_ emissions and the resultant impact on J_A4B4_. These changes to SO_2_ emissions are driven by changes to residential SO_2_ emissions, such as reductions to ash and sulfur content in residential coal. Future emission standards are also expected to drive down SO_2_ from the power sector and these are therefore key in reducing NPF intensity in Beijing.

### Volatility of OOMS

**Figure 4:**
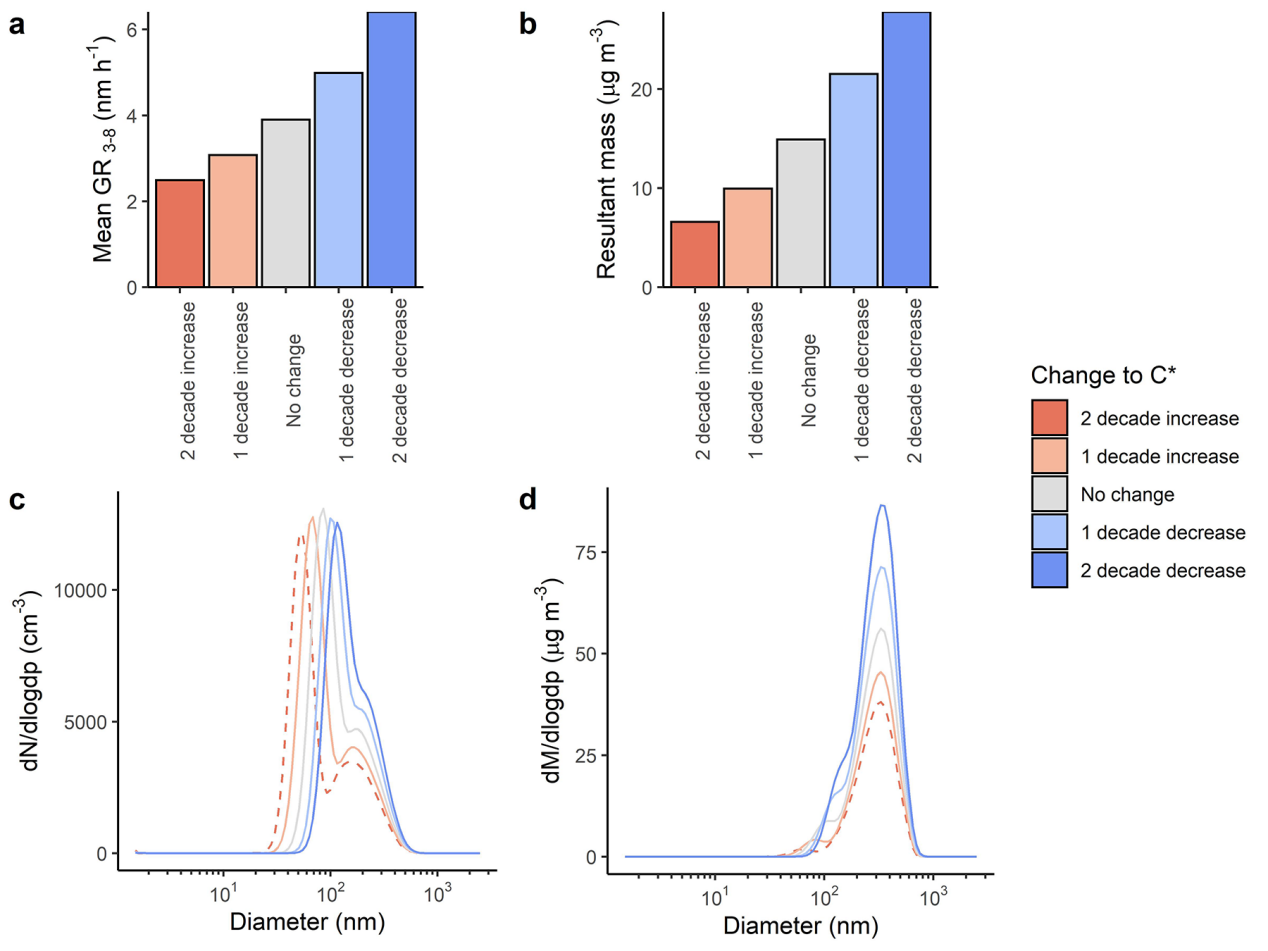
Effect of changing the saturation mass concentration of OOM (μg m^3^) on the Baseline 2020 scenario by 1–2 decades up/down on new particle formation, showing (a) the change to particle growth rates, (b) the change to the particle mass after a 24 h run, (c) the change to the number size distribution at the end of a 24 h run, and (d) the change to the mass size distribution at the end of a 24 h run.

The growth of new particles in Beijing is slow relative to formation when compared to many other environments.^14^ The concentration of OOMs in Beijing is dominated by nitrogen-containing molecules. These have a smaller relative contribution to growth than their non-nitrogen containing counterparts due to their higher saturation mass concentration in the gas phase (C*).^24^ A decrease in NO_*x*_ concentration should theoretically shift the C* of OOMs downward considerably,^46^ but this effect in the real environment is not well-understood. The volatility distribution of OOMs in Beijing used in these calculations, taken from Qiao et al.,^24^ is shown in Figure S1. In our simulations, a greater than 50% decrease to NO_*x*_ emissions is presumed to result in a decrease to OOM volatility of 1 decade in C* based roughly. We further investigate the effect of a shifting volatility distribution of OOMs on NPF and show that growth rates shift by ∼1 nm h^–1^ with a one decade change in saturation mass concentration in our simulations (Figure 4). Yan et al.^46^ show a 40% decrease in GR_3.5–7_ with the addition of 1.9 ppbv NO_*x*_ in a chamber study. We therefore conclude that future decreases to NO_*x*_, and therefore OOM volatility, will result in an enhanced mass of particles arising from NPF presuming all other factors stay the same. A fuller understanding of the effect of NO_*x*_ on the volatility distribution of OOMs is therefore essential to understand future changes to NPF.

### Uncertainties and Future Perspectives

Our estimated future decreases to secondary particle number and mass in China demonstrates a substantial cobenefit of net-zero policy. Alongside changes to PM_2.5_ mass loadings, we also estimate substantial decreases to ultrafine particle count derived from NPF, for which the health burden is currently uncertain.^47^ The initial number and mass concentration of new particles is concentrated in the ultrafine region. By the end of the NPF event, most of the particle mass exists in the accumulation mode however (Figure S8). The CCN enhancement due to NPF in Beijing is large^48^ and greater than other environments.^49^ Changes to the CCN budget due to changes to the currently frequent and intense;^22^ NPF processes may therefore have substantial impacts on the energy balance in the region. Figure S9 shows the diurnal cycle in particle counts, showing substantial changes in the <1000 nm counts. While it is highly likely that multiphase hetereogeneous chemistry is a partial driver of particle growth, especially at sizes greater than 25 nm,^9^ the extent to which this mechanism takes place is also unknown. These simulations of NPF are reliant upon several assumptions. Dimethylamine (DMA) was presumed to be the base solely responsible for particle formation, and the emissions of DMA are presumed to scale with those of NH_3_. Particle formation in Beijing and similar cities has indeed been shown to be driven by DMA;^20,23^ however, should DMA concentrations decrease, other amines may drive NPF (such as monoethanolamine, an important amine during carbon capture^50^ for which we provide estimates of J_A4B4_ in Figure S2), or trimethylamine, which has recently been shown by source apportionment to have distinct sources from DMA.^51^ All VOCs are presumed to scale equally, both those capable and incapable of forming and growing new particles. No other compounds (such as HNO_3_) are considered to contribute to the particle growth. The effects of reducing NO_*x*_ on the oxidation chemistry of OOMs is uncertain, but it is very likely that reductions in NO_*x*_ will reduce the average volatility of OOMs. This could increase particle GRs more substantially than we estimate and thus the particle mass arising from NPF, especially if CSs are to decrease, and therefore particle survival probabilities are to increase (Figure 4). The initial size distribution is key as it determines the CS during NPF, and the shape of this distribution is dependent upon future changes to particle sources, which are also currently unknown but estimated in this work. Should changes to the condensation sink be substantially different then J_A4B4_, GR, and particle survival rates from this work will differ substantially also (Figure S4). Sulfuric acid in Beijing has been shown to be relatively stable as SO_2_ has decreased synchronously with CS,^52^ emphasizing the delicate balance of sources and sink of NPF precursor. An unaccounted-for aspect of the future Beijing atmosphere is the atmospheric oxidation capacity, which will likely also change in the future, but has so far thought to play a minor role in modulating sulfuric acid concentrations relative to CS.^52^ OOMs provide an even more challenging problem in this regard, given the dependence of OOM volatility on condensational loss. We do not account for these changes, and should they be significant, a model reevaluation will be necessary. Finally, SO_2_, NO_*x*_, NH_3_, and VOCs are presumed to have relatively short atmospheric lifespans, and thus, their concentrations are presumed to be tightly coupled with emission rates,^35^ and so, reductions in emissions in the DPEC model are presumed to correspond linearly with reductions to concentrations. We emphasize, therefore, that better understanding of amine concentrations and sources through expanded measurements and source apportionment studies and a better understanding of the future changes to speciated VOCs and particles from different sources as measured in the particle size distribution are key to a more quantitative understanding of future changes to atmospheric NPF.

## Data Availability

Data supporting this publication are openly available from the UBIRA eData repository at 10.25500/edata.bham.00000870.

## Supporting Information Available

Discussion of materials and methods used and figures of volatility distribution of OOM, testing of formation rate simulations, dependence of growth rate on particle diameter, effect of multiplying the particle count across all bins in the initial size distribution, changing temperature, changing VOC concentration, and changing SO_2_ concentration on the Baseline 2020 scenario, particle mass distribution of particles, and diurnal variation in particle number concentration (PDF)

## Abbreviations Used

CS: condensation sink
DPEC: dynamic projection for emission in China
GHG: greenhouse gases
GR: growth rate
J_A4B4_: formation rate
VOCs: volatile organic compounds
