# Supplementary material for: Estimates of Future New Particle Formation under Different Emission Scenarios in Beijing
Source: Environ Sci Technol. 2023 Mar 17;57(12):4741–50. doi: 10.1021/acs.est.2c08348 (PMC10061929; doi:10.1021/acs.est.2c08348)
Supplement: Supplementary file 1 — es2c08348_si_001.pdf [file es2c08348_si_001.pdf]

SUPPLEMENTARY INFORMATION

Estimates of future new particle formation under different emission scenarios in Beijing
James Brean, Alex Rowell, David C.S. Beddows, Zongbo Shi and Roy M. Harrison*

* To whom correspondence should be addressed
Email: r.m.harrison@bham.ac.uk

Number of pages: 17
Number of figures: 9

CONTENTS
Section S1. Materials and Methods
Section S2. References

FIGURES
Fig. S1. Volatility distribution of OOM taken from Qiao et al.[1] input into the script.

Fig. S2. Testing of formation rate simulations of sulphuric acid with 10^7 cm^{-3} DMA, MEA, and NH_3, showing (a) the dependence of J_{A4B4} on temperature with 10^7 cm^{-3} sulphuric acid, 0.01 s^{-1} CS, and 50% RH, (b) the dependence of J_{A4B4} on CS with 10^7 cm^{-3} sulphuric acid, 278 K temperature, and 50% RH, and (c) dependence of J_{A4B4} on sulphuric acid concentrations, with 278 K temperature, 0.01 s^{-1} CS, and 50% RH. Also plotted are $J_{1.7}$ values from chamber studies performed at 278 K and 50 % RH as points, chamber data from Almeida et al.[2] and Kürten et al.[20]

Fig. S3. The dependence of growth rate on particle diameter for (grey) sulphuric acid vapour) and (coloured) OOM of different $\log_{10}(C^*)$ deciles. Data were taken from the Baseline 2020 run at midday, where particle growth rates are highest.

Fig. S4. Effect of multiplying the particle count across all bins in the initial size distribution on the Baseline 2020 scenario, showing the effect on (a) the initial condensation sink, (b) the initial size distribution, (c) the mean $GR_{3\text{-}8}$, (d) the J_{A4B4}, and (f) the particle counts. Here, 1E-1 refers to a multiplication of 10^{-1} to all bins in the initial size distribution.

Fig. S5. Effect of changing temperature on the Baseline 2020 scenario, showing the effect on (a) the mean $GR_{3\text{-}8}$, (b) the J_{A4B4}, (c) the <100 nm particle counts, and (d) the evaporation rate of H_2SO_4-DMA (A_1B_1) clusters.

Fig. S6. Effect of changing VOC concentration on the Baseline 2020 scenario, showing the effect on (a) the OOM concentration, (b) the mean $GR_{3\text{-}8}$, (c) the J_{A4B4}, (d) <100 nm particle counts.

Fig. S7. Effect of changing SO_2 concentration on the Baseline 2020 scenario, showing the effect on (a) the OOM concentration, (b) the mean $GR_{3\text{-}8}$, (c) the J_{A4B4}, (d) <100 nm particle counts.

Fig S8. Particle mass distribution of particles at the end of each model run. Conditions are identical as those for Figure 3.

Fig. S9. Diurnal variation in particle number concentration for <10 nm, <100 nm, and <1,000 nm fractions.

S.1 MATERIALS AND METHODS

We constructed a script simulating new particle formation and growth across a NPF day. The script simulates the formation of sulphuric acid and OOMs. The rate of formation of these particles is simulated by estimating the formation of clusters with 4 acid and 4 base molecules (referred to as J_{A4B4}). Growth of molecules is due to both condensation of acid and base clusters and OOMs, where the volatility distribution of OOMs was taken to be the same as that observed in Beijing [1] (Fig. S1). The concentrations and diurnal profiles of these species, and the resultant formation and growth rates of new particles are similar to those in Beijing.

OH and O_3 concentrations were taken to be similar to those reported previously for Beijing [2, 3] Particles exist in 100 bins between 1.5 and 2500 nm. All simulations were performed at 293 K and 50 % RH. The model runs through 1,440 1-minute timesteps for 24-hours. Sensitivity tests for temperature, CS, OOMs and H_2SO_4 concentration are provided in Fig. S4 through Fig. S7. Particles are formed with 4 acid and 4 base molecules from sulphuric acid and a base molecule with the same properties as DMA, accounting for collisional formation, and losses due to evaporation and coagulation into larger particles.

The DPEC model was used to generate total emission estimates for NH_3, SO_2, VOCs and NO_x in Beijing for the base year (2020) and future years (2040/2060). The climate constraints, socio-economic drivers, and air pollution control measures are summarized in Table 2. Total emissions were used instead of pollutant concentrations due to the availability of data. However, pollutants with relatively short atmospheric lifespans are tightly coupled with emission rates, and therefore fluctuations are reflected in their concentrations[4]. Further documentation and access to the DPEC model is available at http://meicmodel.org, and in refs[5, 6].

The initial size distribution for the base case scenario is taken from the mean size distribution from TSI SMPS (TSI 3080 EC, 3082 Long DMA, 3775 CPC, TSI, USA) data measured in Beijing in the

summertime [7]. For each subsequent scenario, the size distribution was scaled so that the condensation sink increased proportionally to the expected changes to $PM_{2.5}$ mass. Deng et al.[8] find a strong correlation (r = 0.75) between $PM_{2.5}$ mass concentration and condensation sinks.

Photochemistry of the model is driven by OH and O_3 concentrations replicating summertime diurnal cycles [3]. These are consistent between scenarios. SO_2 in the model is oxidised by OH to produce H_2SO_4 according to the proxy of Mikkonen et al.[9]. A model organic molecule is oxidised by both OH and O_3, according to the following equation:

$$[OOM] = \frac{([Org]\cdot[OH]\cdot k_{OH}) + ([Org]\cdot[O_3]\cdot k_{O3})}{CS}$$

Where *CS* is the condensation sink, *Org* is the organic molecule, and k_{OH} *and* k_{O3} are reaction rate constants for HOM formation, here adjusted to give a 2% yield at typical atmospheric conditions for Beijing, in line with typical OOM yields[10]. These OOM are then binned into 15 volatility bins modelled after the volatility distribution of OOMs in springtime Beijing as calculated from the molecular formulae as measured by NO_3^- CIMS. The species were presumed to either arise from autoxidation, and therefore contain mostly hydroperoxide groups, or multi-generational OH oxidation, therefore containing mostly hydroxyl and carboxylic acid groups, depending on their O:C and H:C ratios[1]. Equations following those proposed by Donahue et al.[11] were then used to estimate saturation vapour concentration. The condensation sink, or loss rate of particles is calculated thus [12]:

$$CS = 2\pi D \sum_{d_p} \beta_{m,d_p} d_p N_{d_p}$$

Where D is the diffusion coefficient of the diffusing vapour. Here, the diffusion coefficient of sulphuric acid is used for all molecules. β_m is a transition regime correction, d_p is particle diameter, and N_{dp} is the number of particles at diameter d_p.

Molecular cluster formation was not explicitly modelled, instead, the formation rate of a cluster of 4 acid and 4 base molecules was calculated as according to the following equation [13].

$$J_{A4B4} = \frac{{\beta_{(A1B1)2}}^{3}}{2(CS + \beta_{A1B1}[H_2SO_4])^2} \cdot [H_2SO_4]^4 \cdot \eta^4 \cdot \left[1 + 2 \cdot \frac{\gamma + CS}{\beta_{A1B1} \cdot [B] + CS}\right] \cdot \left[1 + \frac{1}{4} \cdot (1 + 2 \cdot \frac{\gamma + CS}{\beta_{A1B1} \cdot [B] + CS})\right]$$

Where

$$\eta = \frac{\beta[B]}{\gamma + CS + \ \beta_{A1B1}[B]}$$

And where B represents the base molecule, $\beta_{(A1B1)2}$ is the collision coefficient between two clusters containing one acid and one base molecule, and γ is the temperature dependent evaporation rate of a cluster containing one acid and one base molecule (in s^{-1}). In the model, this is treated as the formation rate of particles in the smallest size bin (1.5 nm). The evaporation rate γ is calculated according to

$$\gamma = \frac{\beta_{A1B1} \cdot P}{k_B \cdot T} \cdot e^{\Delta_f G_{m,A1B1}{}^{\theta} / RT}$$

where P is pressure, k_B is Boltzmann's constant, $\Delta_f G_{m,A1B1}{}^{\theta}$ is the Gibbs free energy of formation of a cluster containing one acid and one base, and R is the molar gas constant. $\Delta_f G_{m,A1B1}{}^{\theta}$ at temperature T is calculated from the Gibbs Function, presuming that formation enthalpies and entropies change insubstantially over the experimental temperature range. Formation enthalpies and entropies for acid-base clusters are taken from the literature: Paasonen et al. [14] for sulphuric acid-dimethylamine clusters, Kürten et al. [15] for sulphuric acid-ammonia clusters, and Xie et al. [16] for sulphuric acid-monoethanolamine clusters. The growth of particles is driven by the condensation of both oxygenated organic molecules and sulphuric acid. Growth due to an organic molecule is calculated:

$$GR = (\frac{d_p + d_i}{d_p})^2 \cdot \frac{\bar{c}_{i,p}}{2 \cdot \rho_p} \cdot \alpha_{i,p} \cdot \beta_{m,d_p}[C_i^v - a_{i,p}C_i^0]$$

Where d_p is the diameter of the particle, d_i is the diameter of the vapour, $\bar{c}_{i,p}$ is the mean relative thermal velocity of the gas and particle, ρ_p is the density of particle, here presumed to be 1.5 g cm^{-3}, $\alpha_{i,p}$ is the accommodation coefficient (here presumed to be 1), β_m is a transition regime correction, $a_{i,p}$ is particle phase activity, C_i^v is the vapour phase concentration of *i, and* C_i^0 is the saturation concentration of vapour *i*. $\bar{c}_{i,p}$ is calculated by

$$\bar{c}_{i,p} = \sqrt{\frac{8 \cdot R \cdot T \cdot (M_i + M_p)}{\pi \cdot M_i \cdot M_p}}$$

where M_i and M_p are the molar mass of the gas and particle, respectively. $a_{i,p}$ is calculated as

$$\alpha_{i,p} = y_{i,p} \cdot K_{i,p} \cdot X_{i,p}$$

$y_{i,p}$ is the mass based activity coefficient, here presumed to be unity. $K_{i,p}$ is the Kelvin coefficient. $K_{i,p}$ is equal to

$$K_{i,p} = 10^{D_{K10}/d_p}$$

where D_{K10} is calculated thus

$$D_{K10} = log_{10}(e) \times \frac{4\sigma_i M_i}{RT\rho_i}$$

where σ_i is the surface tension of molecule *i,* here taken as the surface tension of oxidised α-pinene derived particles, 0.044 N m^{-1} [17]. M_i is the mass of molecule *i*, and ρ_i is the density of molecule *i*. $X_{i,p}$ is the mass fraction of vapour *i* in the particle phase

$$X_{i,p} = \frac{C_{i,p}^s}{C_p^s}$$

where $C_{i,p}^{s}$ is the mass of vapour i in the particle phase, and C_{p}^{s} is the sum of $C_{i,p}^{s}$ for all vapours. This was calculated for each size bin and vapour in the model for each timestep. The coagulation coefficient in the free molecular regime is calculated as follows[18]:

$$k_{j,l} = (\frac{3}{4\pi})^{1/6} \cdot \sqrt{\frac{6k_B T}{\rho_p} \cdot \left(\frac{1}{v_j} + \frac{1}{v_l}\right)}^{1/2} \cdot ({v_j}^{1/3} + {v_l}^{1/3})^2$$

For each timestep, particles in each size bin coagulate into all larger size bins iteratively, resulting in a decrease in particle number with a maintenance in particle volume.

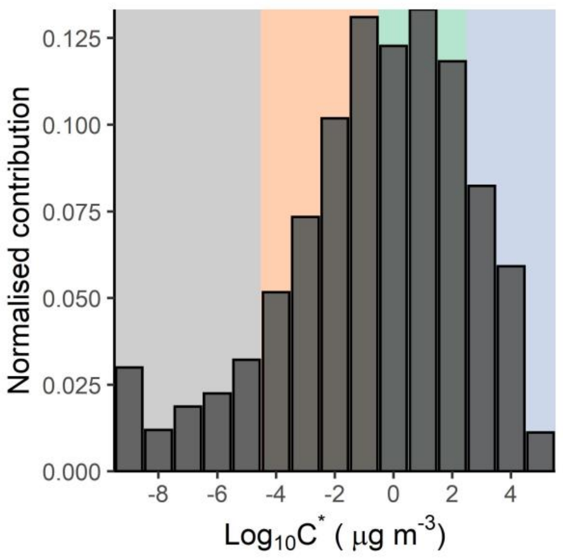

Fig. S1. Volatility distribution of OOM taken from Qiao et al.[1] input into the script.

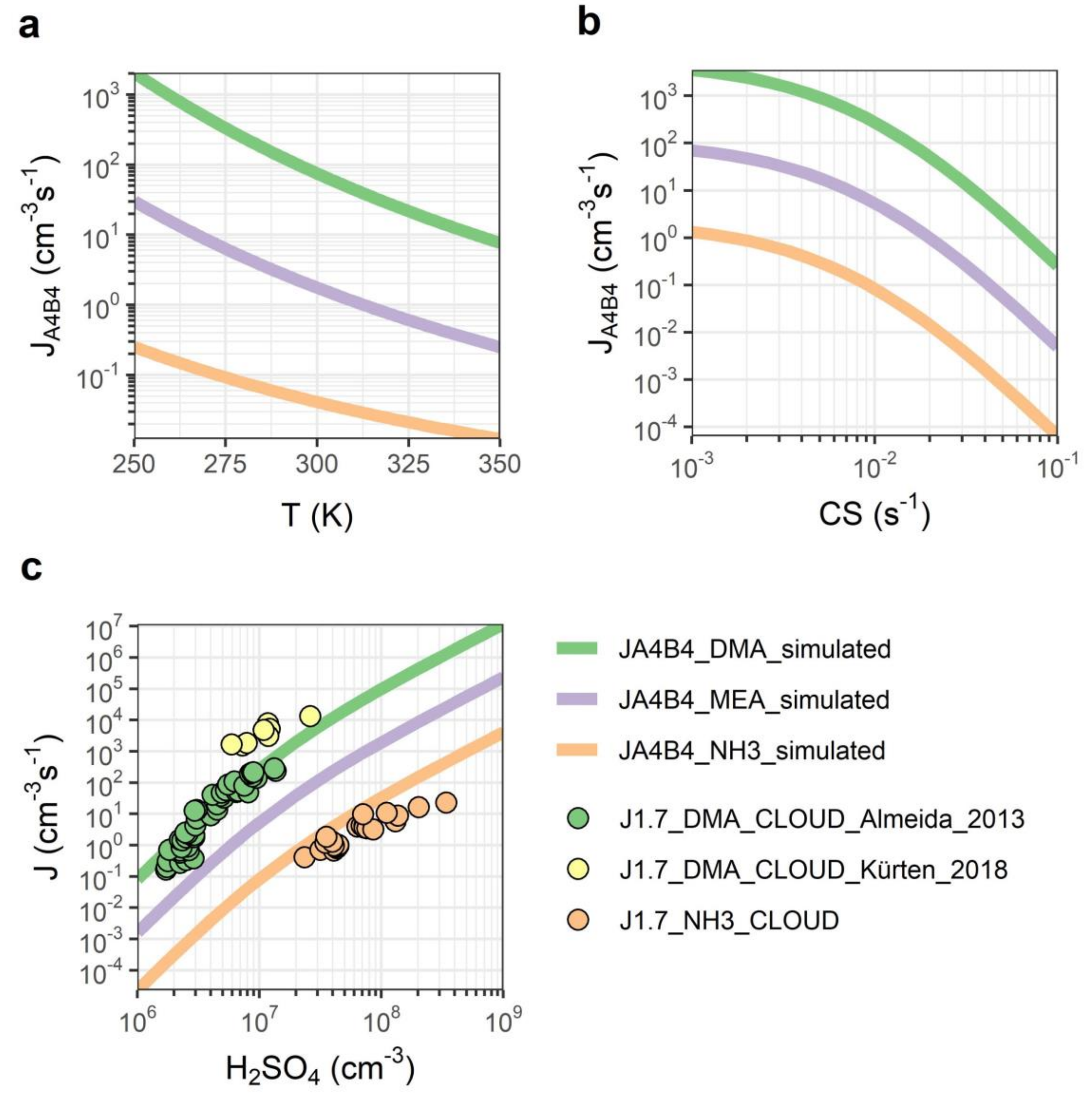

Fig. S2. Testing of formation rate simulations of sulphuric acid with 10^7 cm^{-3} DMA, MEA, and NH_3, showing (a) the dependence of J_{A4B4} on temperature with 10^7 cm^{-3} sulphuric acid, 0.01 s^{-1} CS, and 50% RH, (b) the dependence of J_{A4B4} on CS with 10^7 cm^{-3} sulphuric acid, 278 K temperature, and 50% RH, and (c) dependence of J_{A4B4} on sulphuric acid concentrations, with 278 K temperature, 0.01 s^{-1} CS, and 50% RH. Also plotted are $J_{1.7}$ values from chamber studies performed at 278 K and 50 % RH as points, chamber data from Almeida et al.[19] and Kürten et al.[20]

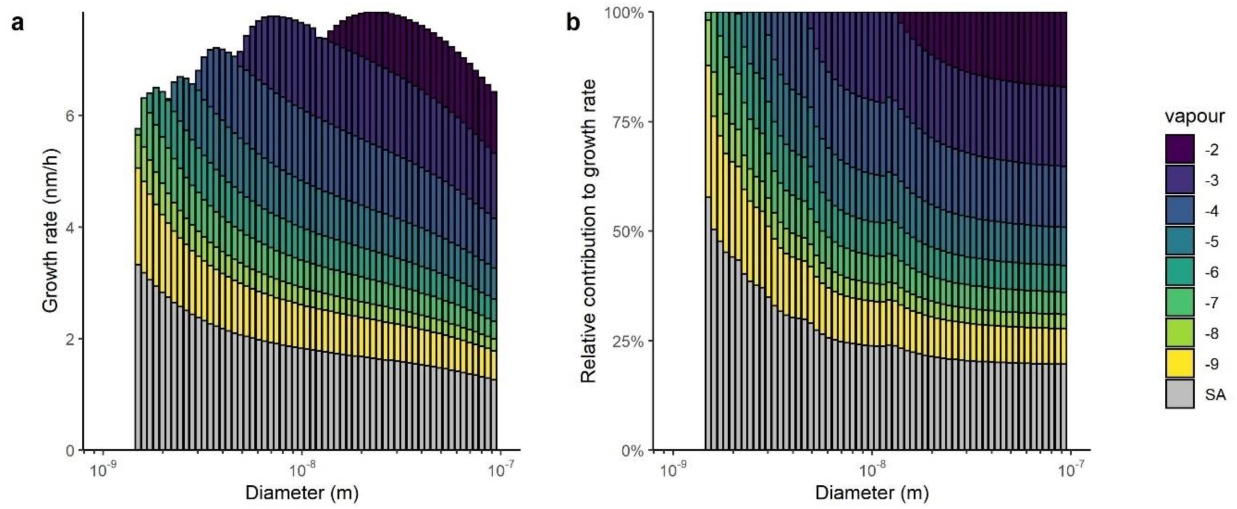

Fig. S3. The dependence of growth rate on particle diameter for (grey) sulphuric acid vapour) and (coloured) OOM of different $\log_{10}(C^*)$ deciles. Data were taken from the Baseline 2020 run at midday, where particle growth rates are highest.

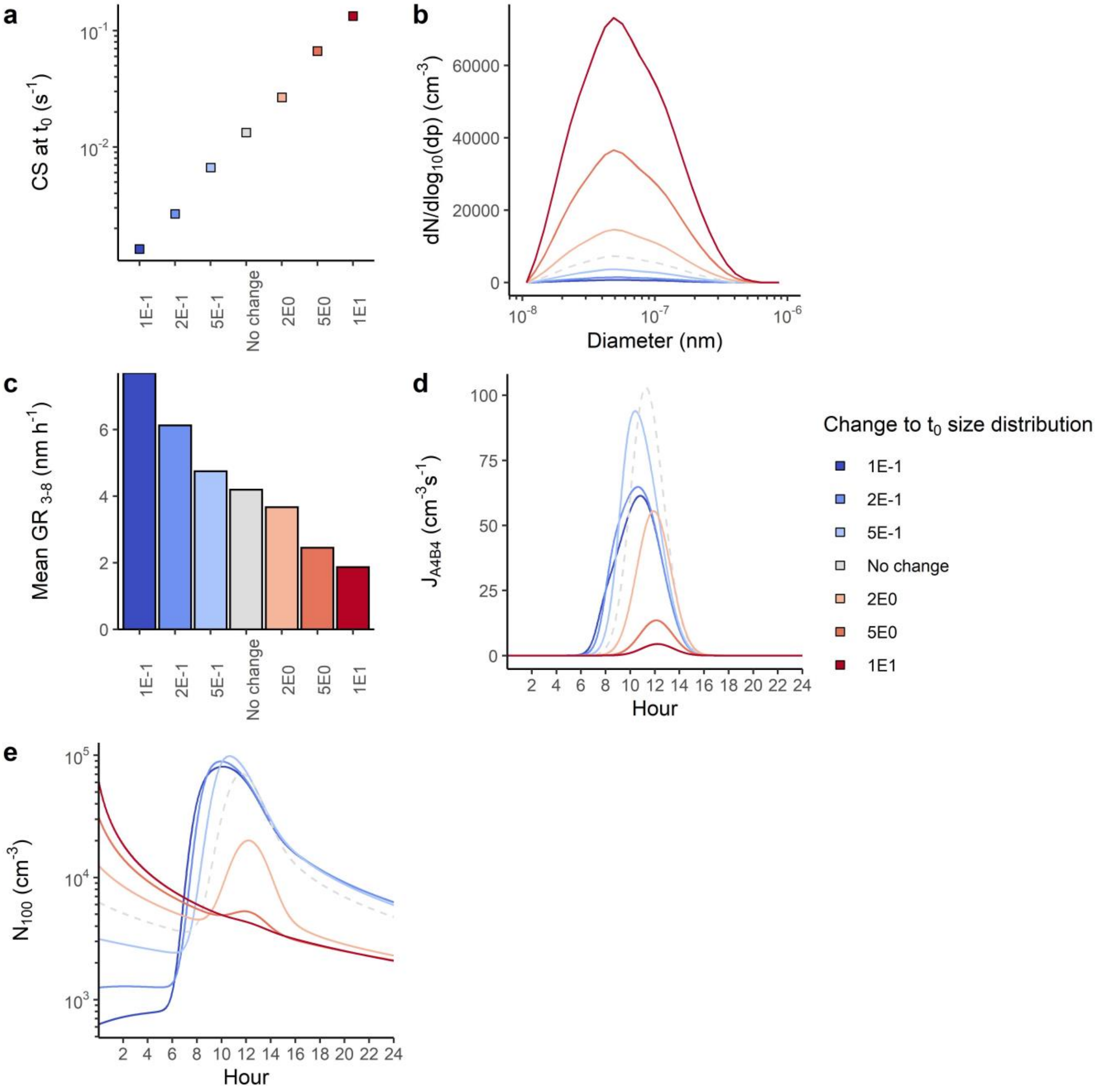

Fig. S4. Effect of multiplying the particle count across all bins in the initial size distribution on the Baseline 2020 scenario, showing the effect on (a) the initial condensation sink, (b) the initial size distribution, (c) the mean $GR_{3\text{-}8}$, (d) the J_{A4B4}, and (f) the particle counts. Here, 1E-1 refers to a multiplication of 10^{-1} to all bins in the initial size distribution.

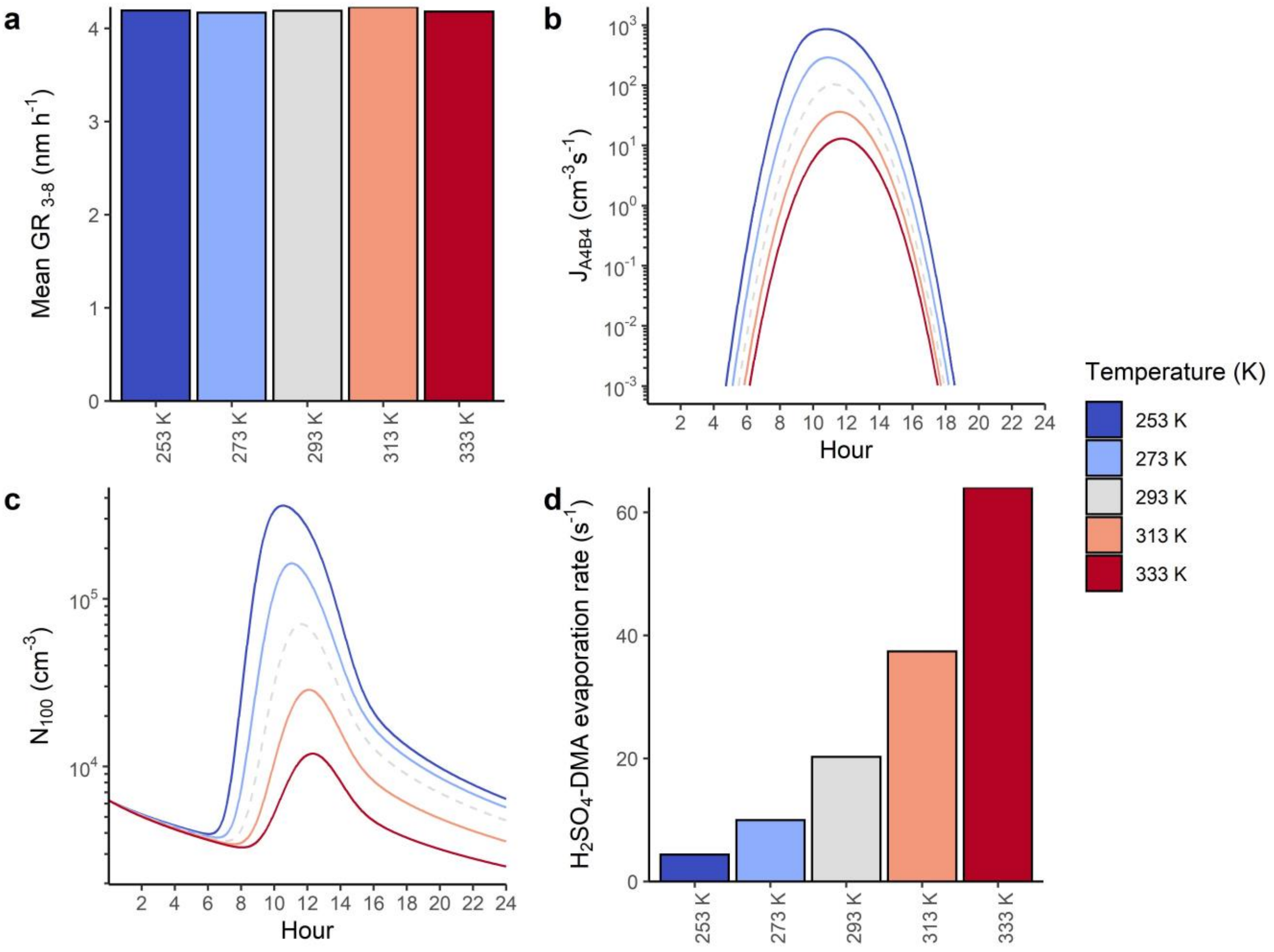

Fig. S5. Effect of changing temperature on the Baseline 2020 scenario, showing the effect on (a) the mean GR_{3-8}, (b) the J_{A4B4}, (c) the <100 nm particle counts, and (d) the evaporation rate of H_2SO_4-DMA (A_1B_1) clusters.

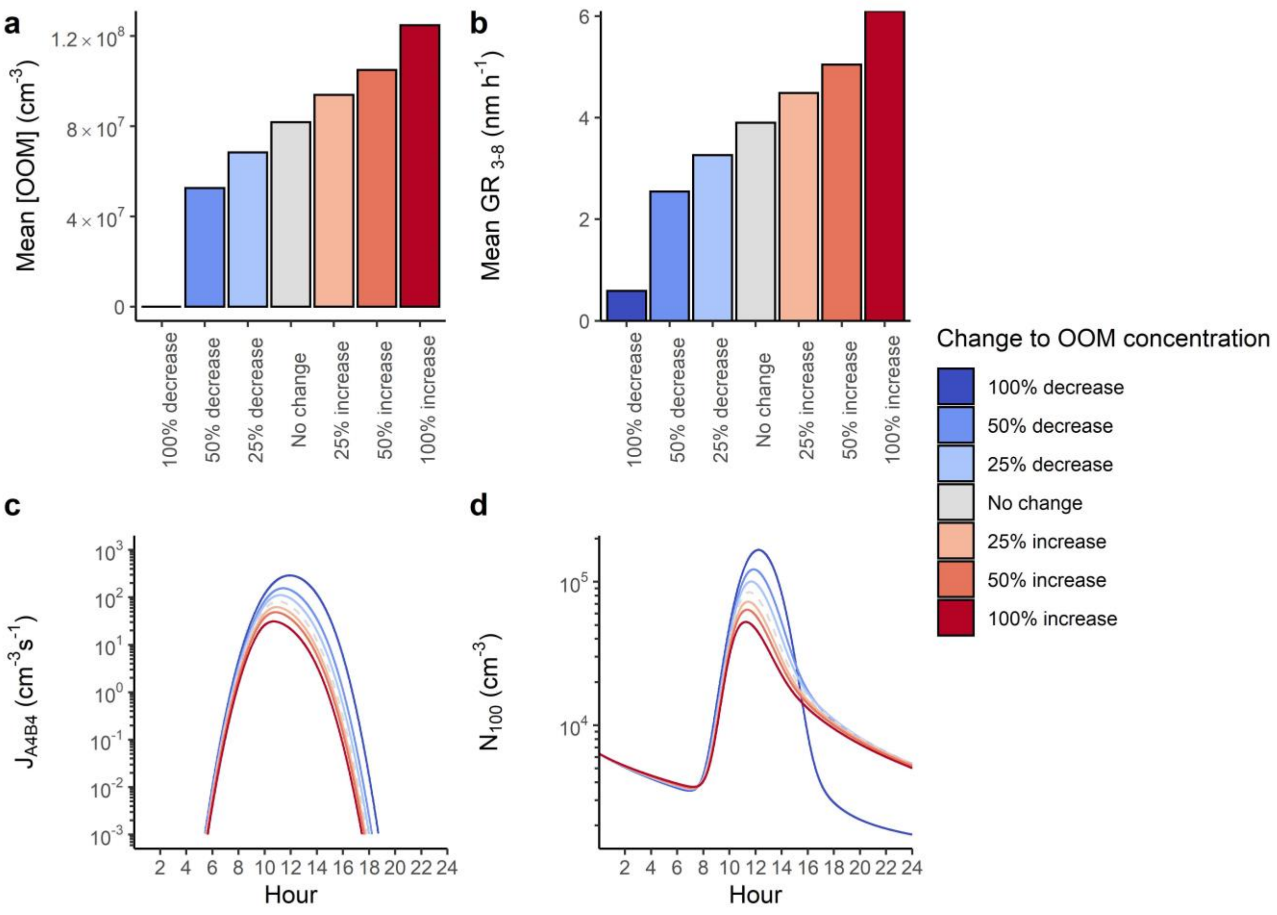

Fig. S6. Effect of changing VOC concentration on the Baseline 2020 scenario, showing the effect on (a) the OOM concentration, (b) the mean GR_{3-8}, (c) the J_{A4B4}, (d) <100 nm particle counts.

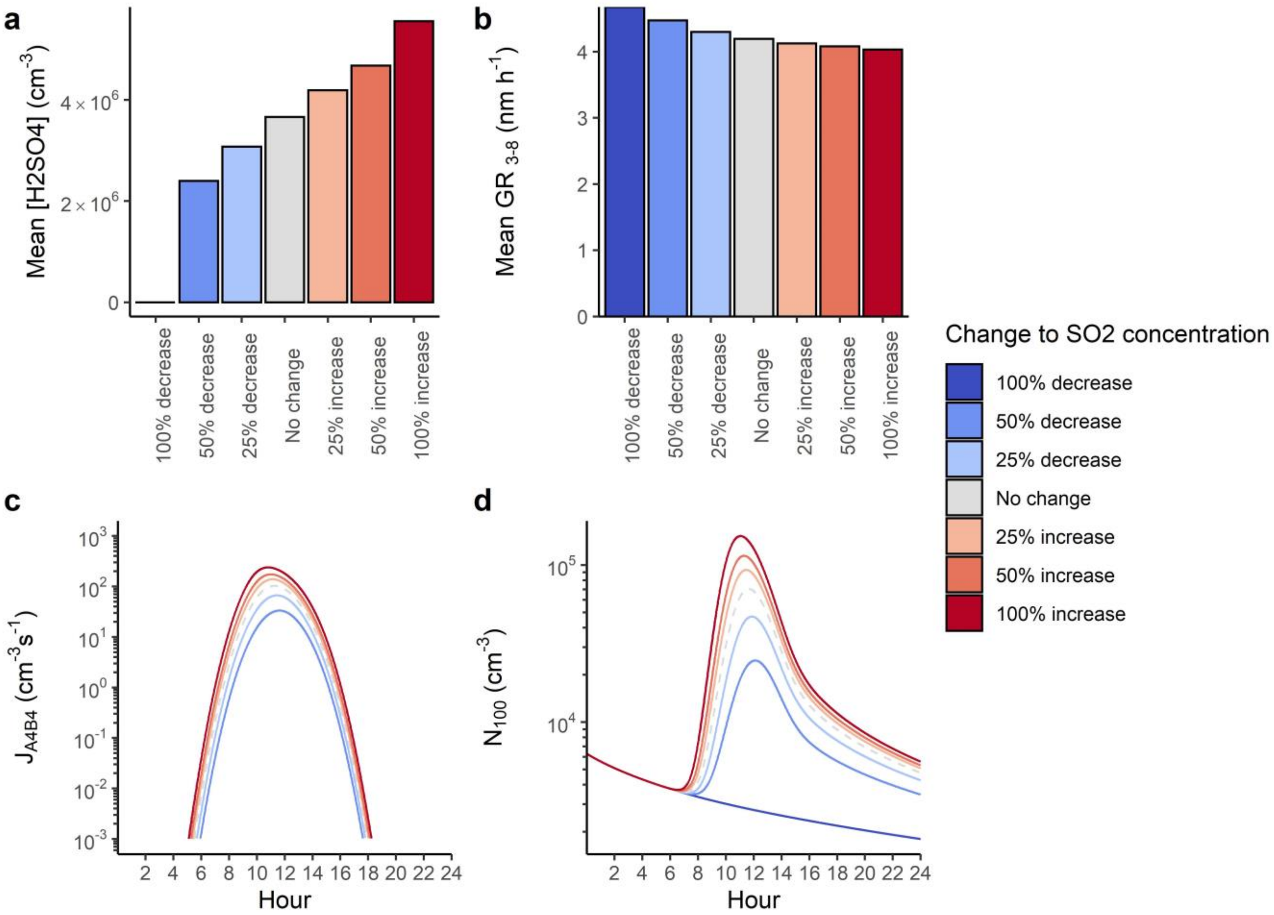

Fig. S7. Effect of changing SO_2 concentration on the Baseline 2020 scenario, showing the effect on (a) the H_2SO_4 concentration, (b) the mean $GR_{3\text{-}8}$, (c) the J_{A4B4}, (d) <100 nm particle counts.

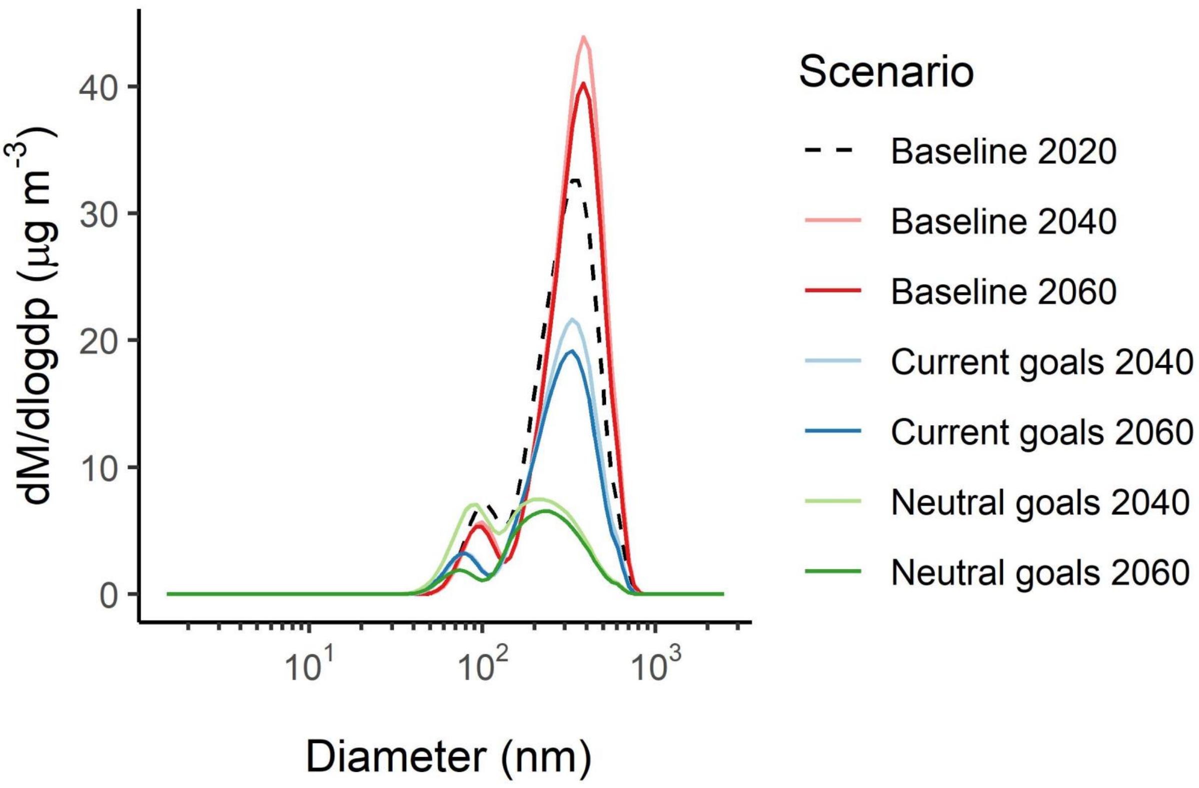

Fig S8. Particle mass distribution of particles at the end of each model run. Conditions are identical as those for Figure 3.

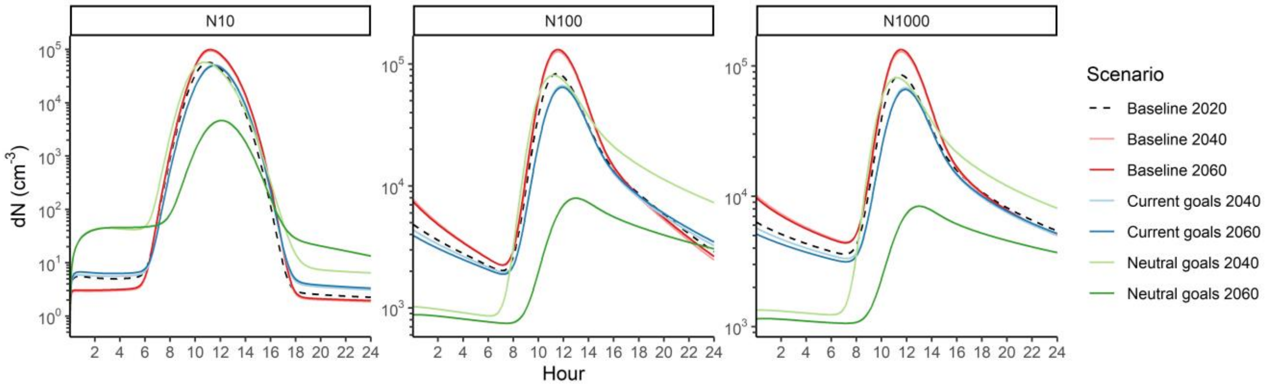

Fig. S9. Diurnal variation in particle number concentration for <10 nm, <100 nm, and <1,000 nm fractions.

REFERENCES

(1) Qiao, X.; Yan, C.; Li, X.; Guo, Y. S.; Yin, R.; Deng, C.; Li, C.; Nie, W.; Wang, M.; Cai, R.; et al. Contribution of Atmospheric Oxygenated Organic Compounds to Particle Growth in an Urban Environment. *Environmental Science and Technology* **2021**, *55*, 13646-13656. DOI: 10.1021/acs.est.1c02095.
(2) Shi, Z.; Vu, T.; Kotthaus, S.; Roy, M. H.; Grimmond, S.; Yue, S.; Zhu, T.; Lee, J.; Han, Y.; Demuzere, M.; et al. Introduction to the special issue "In-depth study of air pollution sources and processes within Beijing and its surrounding region". *Atmospheric Chemistry and Physics* **2019**, *19* (11), 7519-7546.
(3) Whalley, L.; Slater, E.; Woodward-Massey, R.; Ye, C.; Lee, J.; Squires, F.; Hopkins, J.; Dunmore, R.; Shaw, M.; Hamilton, J.; et al. Evaluating the sensitivity of radical chemistry and ozone formation to ambient VOCs and NOx in Beijing. *Atmospheric Chemistry and Physics* **2021**, *21*, 2021-2147. DOI: 10.5194/acp-2020-785.
(4) Council, N. R. *Climate Stabilization Targets: Emissions, Concentrations, and Impacts over Decades to Millennia*; 2011. DOI: 10.17226/12877.
(5) Tong, D.; Cheng, J.; Liu, Y.; Yu, S.; Yan, L.; Hong, C.; Qin, Y.; Zhao, H.; Yixuan, Z. Dynamic projection of anthropogenic emissions in China: methodology and 2015 – 2050 emission pathways under a range of socio-economic, climate policy, and pollution control scenarios. *Atmospheric Chemistry and Physics* **2020**, *20* (9), 5729-5757.
(6) Cheng, J.; Tong, D.; Liu, Y.; Yu, S.; Yan, L.; Zheng, B.; Geng, G.; He, K.; Zhang, Q. Comparison of Current and Future PM2.5 Air Quality in China Under CMIP6 and DPEC Emission Scenarios. *Geophysical Research Letters* **2021**, *48*, 1-11. DOI: 10.1029/2021GL093197.
(7) Brean, J.; Harrison, R. M.; Shi, Z.; Beddows, D. C. S.; Acton, W. J. F.; Hewitt, C. N.; Squires, F. A.; Lee, J. Observations of highly oxidized molecules and particle nucleation in the atmosphere of Beijing. *Atmospheric Chemistry and Physics* **2019**, *19*. DOI: 10.5194/acp-19-14933-2019.
(8) Deng, C.; Cai, R.; Chao, Y. Formation and growth of sub-3 nm particles in megacities: impact of background aerosols. *Faraday Discussions* **2021**, 348-363. DOI: 10.1039/d0fd00083c.
(9) Mikkonen, S.; Romakkaniemi, S.; Smith, J. N.; Korhonen, H.; Petäjä, T.; Plass-Duelmer, C.; Boy, M.; McMurry, P. H.; Lehtinen, K. E. J.; Joutsensaari, J.; et al. A statistical proxy for sulphuric acid concentration. *Atmospheric Chemistry and Physics* **2011**, *11*, 11319-11334. DOI: 10.5194/acp-11-11319-2011.
(10) Federico Bianchi and Olga Garmash and Xucheng He and Chao Yan and Siddharth Iyer and Ida Rosendahl and Zhengning Xu and Matti, P. R. a. M. R. a. R. T. a. The role of highly oxygenated molecules (HOMs) in determining the composition of ambient ions in the boreal forest. *Atmospheric Chemistry and Physics* **2017**, *17*, 13819-13831. DOI: 10.5194/acp-17-13819-2017.
(11) Donahue, N. M.; Epstein, S. A.; Pandis, S. N.; Robinson, A. L. Atmospheric Chemistry and Physics A two-dimensional volatility basis set: 1. organic-aerosol mixing thermodynamics. *Atmos. Chem. Phys* **2011**, *11*, 3303-3318. DOI: 10.5194/acp-11-3303-2011.
(12) Kulmala, M.; Petäjä, T.; Nieminen, T.; Sipilä, M.; Hanna, E. M.; Lehtipalo, K.; Maso, M. D.; Aalto, P. P.; Junninen, H.; Paasonen, P.; et al. Measurement of the nucleation of atmospheric aerosol particles. *Nature Protocols* **2012**, *7*, 1651-1667. DOI: 10.1038/nprot.2012.091.
(13) Cai, R.; Yan, C.; Douglas, R. W.; Bianchi, F.; Kerminen, V. M.; Liu, Y.; Wang, L.; Zheng, J.; Kulmala, M.; Jiang, J. An indicator for sulfuric acid–amine nucleation in atmospheric environments. *Aerosol Science and Technology* **2021**, *55*, 1059-1069. DOI: 10.1080/02786826.2021.1922598.
(14) Paasonen, P.; Olenius, T.; Kupiainen, O.; KurtÃn, T.; Petäjä, T.; Birmili, W.; Hamed, A.; Hu, M.; Huey, L. G.; Plass-Duelmer, C.; et al. On the formation of sulphuric acid – Amine clusters in varying atmospheric conditions and its influence on atmospheric new particle formation. *Atmospheric Chemistry and Physics* **2012**, *12*, 9113-9133. DOI: 10.5194/acp-12-9113-2012.
(15) Andreas, K. New particle formation from sulfuric acid and ammonia: Nucleation and growth model based on thermodynamics derived from CLOUD measurements for a wide range of conditions. *Atmospheric Chemistry and Physics* **2019**, *19*, 5033-5050. DOI: 10.5194/acp-19-5033-2019.
(16) Xie, H. B.; Elm, J.; Halonen, R.; Myllys, N.; Kurtén, T.; Kulmala, M.; Hanna, V. Atmospheric Fate of Monoethanolamine: Enhancing New Particle Formation of Sulfuric Acid as an Important Removal Process.

Environmental Science and Technology **2017**, *51*, 8422-8431 , pmid = 28651044. DOI: 10.1021/acs.est.7b02294.
(17) Andrew, D. H.; Raymond, T. M.; Dutcher, D. D. A method for the direct measurement of surface tension of collected atmospherically relevant aerosol particles using atomic force microscopy. *Atmospheric Chemistry and Physics* **2016**, *16*, 9761-9769. DOI: 10.5194/acp-16-9761-2016.
(18) Lee, K. W.; Chen, H. Coagulation Rate of Polydisperse Particles. *Aerosol Science and Technology* **1984**, *3* (3), 327-334. DOI: 10.1080/02786828408959020
(19) Almeida, J.; Schobesberger, S.; Kürten, A.; Ismael, K. O.; Kupiainen-Määttä, O.; Praplan, A. P.; Adamov, A.; Amorim, A.; Bianchi, F.; Breitenlechner, M.; et al. Molecular understanding of sulphuric acid-amine particle nucleation in the atmosphere. *Nature* **2013**, *502*, 359-363. DOI: 10.1038/nature12663.
(20) Kürten, A.; Li, C.; Bianchi, F.; Curtius, J.; Dias, A.; Neil, M. D.; Duplissy, J.; Flagan, R. C.; Hakala, J.; Jokinen, T.; et al. New particle formation in the sulfuric acid-dimethylamine-water system: Reevaluation of CLOUD chamber measurements and comparison to an aerosol nucleation and growth model. *Atmospheric Chemistry and Physics* **2018**, *18*, 845-863. DOI: 10.5194/acp-18-845-2018.
